# 

**Published:** 1988

**Authors:** 


**Editor**
Mr M. G. Wilson
*40 Coombe Lane, Bristol BS9 2BJ*
*Tel: 0272-682320*

**Assistant Editor**
Dr P. R. Goddard

**Editorial Committee**
Mr B. P. Jones (Secretary),
*Medical Librarian, Bristol University*
Dr J. L. Burton

**Correspondents**
Dr A. E. Adam (Taunton)
Mr D. C. C. Bartolo
Mr M. L. Crosfill (Penzance)
Dr J. D. Davies
Dr J. Jancar
Professor D. J. Pereira Gray (Exeter)
Dr H. G. Mather
Professor G. M. Stirratt
Mr C. Teasdale (Plymouth)
Dr J. L. G. Thomson
Dr M. J. Whitfield
Professor G. K. Wilcock

**BRISTOL MEDICO-CHIRURGICAL SOCIETY**

**President**
Dr H. M. Norman

**Secretary**
Mr R. N. Baird,
*23 Old Sneed Park, Bristol BS9 1RG*

**Treasurer**
Dr N. C. Tricks,
*The Mill, Wrington, Bristol*

**Published by**
**Clinical Press,**
*Greinton House,*
*8 Exeter Buildings,*
*Redland,*
*Bristol BS6 6TH*

**Typesetting by**
*Avon Communications Ltd.,*
*Avon House, Nailsea, Bristol BS19 2DJ*

**Printed by**
*Sutton Print, PaigntonTQ4 7QR*

Established 1883

### BRISTOL MEDICO-CHIRURGICAL JOURNAL

**1988**
**Volume 102 (1a)**

Copyright © Clinical Press Ltd 1988.

Published quarterly and distributed free to all doctors i
the West of England. Overseas subscription £12.

**NOTICE TO CONTRIBUTORS**
Contributions are invited especially from West of England authors on a variety of subjects, e.g. Original articles relating to the practic of medicine, reports on the contemporary medical scene, obituaries, historical accounts, recollections of colleagues and events worth to be remembered and liable to be forgotten. An article of 2,000 words with 2 illustrations will occupy 2 pages and this is a suggeste though by no means an obligatory length. Articles are preferred in the form proposed by the International Committee of Medica Editors (Vancouver System)—pamphlet obtainable from Editor of BMJ., BMA. House, Tavistock Square, London, WC1H 9JR.

### Bristol Medico-Chirurgical Journal

"The Medical Journal of the South West"
Volume 102, (1a) 1988, Special Supplement

### CURRENT THEMES IN PAEDIATRIC ONCOLOGY
### Symposium held in Bristol April 24–25th 1987

**SPEAKERS**

**Dr P. J. Berry, BA, MB, BChir, MRCP, MRCPath**

Consultant Paediatric Pathologist, Bristol Children's Hospital

**Dr Keith Brown, MA, MSc, PhD**

Dept of Pathology, University of Bristol

**Dr E. J. Chambers, MB, BS, MRCS, LRCP, DCH, DObst RCOG**

Clinical Assistant in Paediatric Oncology, Bristol Children's Hospital

**Ms Chris Colburn, MEd**

Hospital Teacher, Bristol Children's and Winford Orthopaedic Hospital

**Dr J. M. Cornish, MB, ChB, DObst RCOG**

Clinical Assistant in Paediatric Oncology, Bristol Children's Hospital

**Dr Jan Culling, MB, BS, MRCPsych**

Consultant Child Psychiatrist, Southmead Hospital, Bristol

**Ms Sally Curnick, SRN, RSCN**

Family Support Sister, Bristol Children's Hospital

**Dr Nicholas Foreman, MB, ChB, MRCP**

CLIC Research Fellow, Bristol Children's Hospital

**Dr John James, MB, ChB, MRCGP**

GP and Clinical Assistant in Paediatric Oncology, Bristol Children's Hospital

**Dr Carol Kendrick BSc, MB, ChB**

Psychologist, Bristol

**Mr Geoffrey Moss, LLB, MEd(Ed Psych), MEd, DCP, ABPS**

Senior Educational Psychologist, Avon

**Dr Martin Mott, BSc, MB, ChB, FRCP, DCH**

Consultant Paediatrician/Oncologist, BCH, Bristol Children's Hospital

**Dr Barry Pizer, MB, ChB, MRCP**

Paediatric Registrar, King's Mill Hospital, Sutton in Ashfield

**Dr Sawson Ritha, MB, ChB, MRCP, DCH**

Research Fellow, Bristol Children's Hospital

**Mr Andrew Shaw, BSc**

Dept of Pathology, University of Bristol

**Dr David Shortland, MB, ChB MRCP**

Research Registrar, Leicester

**Dr Elaine Simpson, MB, ChB, MRCP**

Senior Registrar in Haematology, Great Ormond St. Children's Hospital

**Dr Paul Ward, BSc, MB, ChB, MRCP, DCH**

Senior Registrar, Jenny Lind Children's Dept., Norfolk and Norwich Hospital

**Dr Nigel Weightman, MB, BS**

Senior Registrar in Microbiology, Preston Public Health Laboratory

**Mr James Wisheart, BSc(Hons), MCh(Hons), FRCS Ed, DObst RCOG**

Consultant Cardiothoracic Surgeon, Bristol Children's Hospital

